# Genome Wide Identification of Structure Variations in Five Italian Turkey Populations

**DOI:** 10.3390/ani15030339

**Published:** 2025-01-24

**Authors:** Medhat S. Saleh, Vincenzo Landi, Martijn F. L. Derks, Gerardo Centoducati, Martien A. M. Groenen, Pasquale De Palo, Elena Ciani, Nicola Pugliese, Elena Circella, Antonio Camarda

**Affiliations:** 1 Department of Veterinary Medicine, University of Bari Aldo Moro, 70010 Valenzano, Italy; medhat.elshahat@uniba.it (M.S.S.); gerardo.centoducati@uniba.it (G.C.); pasquale.depalo@uniba.it (P.D.P.); nicola.pugliese@uniba.it (N.P.); elena.circella@uniba.it (E.C.); antonio.camarda@uniba.it (A.C.); 2 Animal Breeding and Genomics, Wageningen University & Research, P.O. Box 338, 6700 AH Wageningen, The Netherlands; martijn.derks@wur.nl (M.F.L.D.); martien.groenen@wur.nl (M.A.M.G.); 3 Department of Animal Production, Faculty of Agriculture, Benha University, Benha 13736, Egypt; 4 Department of Biosciences, Biotechnologies and Environment, University of Bari Aldo Moro, 70125 Bari, Italy; elena.ciani@uniba.it

**Keywords:** structure variations, whole genome sequencing, population genetic structure, turkey

## Abstract

Structural variants (SVs) are one of the main sources of genetic variants and have a significant impact on phenotype evolution, disease susceptibility, and environmental adaptations. We used 73 whole genome sequencing (12x) to apply a mapping approach to identify SVs in five turkey populations. A notable degree of genetic isolation was observed between the Basilicata and Apulian populations, as indicated by principal component analysis and admixture results. A total of 11,733 SVs were detected, including 6712 deletions, 2671 duplications, 1430 inversions, and 920 translocations. The Variant Effect Predictor (VEP) analysis predicted various consequences of filtered SVs as follows: intron variants (35.8%), intergenic variants (9.6%), coding sequence variants (8.3%), downstream gene variants (7.5%), and transcript ablations (7.3%). Our functional annotation of genes overlapping with SVs was mainly enriched in recognized pathways governing positive regulation of nucleoplasm, protein binding, mitochondrion, negative regulation of cell population proliferation, identical protein binding, and calcium signaling. We produced a comprehensive SV catalog utilizing unique whole-genome turkey data. This SV catalog not only increases our understanding of genetic diversity in turkeys but also enhances our knowledge of the role of SVs in their phenotypic traits.

## Simple Summary

Structure variations are believed to be a significant part of evolutionary processes and are crucial factors for phenotypic variations. A total of 73 whole genome sequencing of five Italian turkey populations were used to detect structure variations. A comprehensive structure variations catalog was produced involving deletions, duplications, inversions, and translocations. The annotation of structure variations revealed that intron variants, intergenic variants, coding sequence variants, downstream gene variants, and transcript ablations were the most common structure variation outcomes. This structural variant catalog will help in understanding genetic diversity in turkeys and provide valuable insights into the influence of structure variations on economic traits.

## 1. Introduction

Turkeys are considered one of the most important agricultural species. Turkey meat is the second most popular poultry meat worldwide, and there is significant consumer demand for fresh, lower-fat, protein-rich meat [1]. In 2021, turkey meat production reached 6.1 million tons, representing 4% of the world’s poultry meat and 1.6% of the global meat production [2]. *Meleagris gallopavo* is the genus name used for both domesticated and wild turkeys, and it is currently classified into six subspecies: *M. g. gallopavo*, *M. g. mexicana*, *M. g. intermedia*, *M. g. merriami*, *M. g. silvestris*, and *M. g. osceola* [3]. Turkey domestication began around 1800 BC in southern Mexico and about 200 BC in the American Southwest [4]. Genetic research reveals that the southern Mexican subspecies (*M. g. gallopavo*) is the ancestor of the domestic turkeys now raised worldwide through documented trade between Europe and the Americas in the 16th century [5]. Turkeys were introduced to Italy in 1520, and their rapid spread, particularly in the southern regions, confirms their integration into Italy’s agricultural and culinary heritage [6,7]. Also, bone remains indicate that they were also used for food starting in 1600, to the point that they are described in cooking recipes of the time [6,8].

Indigenous Italian turkey breeds are a source of genetic diversity that must be preserved and exploited. These native turkey breeds have unique production traits, are resistant to disease, and are adapted to local conditions [9,10]. Election by farmers over the past five centuries has led to significant variation in feather color, body size, and weight among Italian turkey breeds, contributing to their differentiation [10,11,12]. These divergences may have also been influenced by Italy’s geopolitical structure, which was divided into numerous small states with limited product and population exchange, resulting in genetic isolation among turkey populations [13]. Basilicata and Apulian turkeys (BAS-APUs) are sustainable in the Basilicata and Puglia regions of southern Italy. They have different plumage colors, buff and black, with streaks of buff or white. The body weight of adult birds is up to 9 and 4 kg for males and females, respectively. The number of eggs is 50/60 egg per year. BAS-APU populations are rustic and climate-adapted to local conditions.

The detection and genotyping of genetic variations provide a foundation for genetic evaluation. Over the past decade, advancements in genomics have enabled the reliable identification of millions of single nucleotide polymorphisms (SNPs) through whole-genome sequencing (WGS) and high-throughput genotyping using SNP panels [14,15]. SNP array data are commonly used in animal breeding to identify SNPs and detect unbalanced structural variants (SVs), such as copy number variants (CNVs), a subgroup of SVs that includes deletions and duplications. However, array-based SV detection has limitations, such as low resolution and undefined breakpoints [16]. Alternatively, short-read WGS data offers a more accurate method for identifying SVs, including CNVs and balanced SVs like inversions [17].

SVs are genetic rearrangements larger than 50 base pairs (bp) and include deletions, insertions, inversions, duplications, translocations, and CNVs [16]. Numerous novel SVs and CNVs have been associated with phenotypic variation, production traits, and immune responses in farm animals, including turkeys [16,18,19], chickens [20,21,22], sheep [23,24], cattle [25,26,27], and pigs [28,29]. While much of the research has focused on CNVs, small insertions or deletions, and SNPs in turkey populations, the study of larger SVs remains limited despite their potential impact on phenotypes. Therefore, the objective of this study is to identify structural variations across five Italian turkey populations using whole-genome sequencing data.

## 2. Materials and Methods

### 2.1. Animals and Sampling

A total of 73 blood samples (about 2 mL) were collected from wing veins of Basilicata (BAS; 46 samples), Apulian M (APU_M; 7 samples), and Apulian PS (APU_PS; 8 samples) in Potenza, Basilicata region and Apulian PN (APU_PN; 7 samples), and Apulian C (APU_C; 5 samples) populations in marginal area of Brindisi and Lecce, Puglia region, Italy (Supplementary File S1: Table S1). Detailed information about these populations and sampling was mentioned in our previous study [30].

### 2.2. Sequencing and Mapping

DNA extraction and whole genome sequencing (coverage  =  12x) were assessed at Neogen (Ayr, Scotland, UK) using a commercial kit. The Burrows–Wheeler Aligner BWA-MEM v0.7.17 [31] was used to align raw reads to a new turkey reference assembly [*Meleagris gallopavo* (Turkey)-GCA_905368555.1 (MGAL_WU_HG_1.0)] [32], generating a BAM file for each animal. These BAM files were sorted and indexed with SAMtools v.1.9 [33]. Duplicate reads were detected and eliminated by the Samtools dedup function [33]. Mapping statistics outputs were generated using Qualimap [34].

### 2.3. Analysis of Population Structure

To indirectly verify the results of the SV detection study, we compared the population structure derived from SVs with those obtained from SNPs. The principal component analysis (PCA) was employed using the PLINK v.1.9 software [35] using all validated SV information and visualized using the R package ggplot2 [36]. The SNP data of our previous manuscript were used to perform PCA for comparison with the PCA of SVs and to perform the admixture genetic analysis [37]. The ADMIXTURE 1.3 genetic analysis software was used to assess the population genome-wide genetic structure among the populations [38], using different numbers of ancestral populations. The most probable number of ancestral populations was identified in conjunction with the lowest cross-validation error (CV), setting the analysis with an optimal number of clusters (K-value) from 2 to 8, and visualized with BITE V2 R package [39].

### 2.4. SVs Calling and Annotation

We identified SVs using the Smoove pipeline (https://github.com/brentp/smoove, accessed on 21 August 2021). Smoove uses different pieces of software to call and filter SVs, using aligned BAM files and the turkey reference genome GCA_905368555.1 (MGAL_WU_HG_1.0) as inputs. In the first step, the Lumpy software was used to call SVs [40]. In the second step, Svtyper was used to combine all SV calls into a single variant call format (VCF) file [41]. In the third step, Svtyper was also used to genotype SVs at the population level. The pipeline generated a VCF file in which all detected SVs were referenced, and each sample was assigned a genotype for each SV. Duphold was used in the fourth step to add depth information, which can be implemented to filter out false-positive SVs [42].

SVs were annotated using BCFtools [43], with the Ensembl rapid release [*Meleagris gallopavo* (Turkey)-GCA_905368555.1 (MGAL_WU_HG_1.0)] annotations.

### 2.5. Post-Filtration and SV Processing

Four types of SVs are discovered with Lumpy: deletions, duplications, inversions, and breakend variants. To reduce the discovery of false-positive SVs further, we used the following criteria: (1) thresholds for the duphold-generated tag-DHFFC- were set to 0.7 and 1.3 for deletion and duplication, respectively, i.e., for deletion, it should be less than 0.7, while, for duplication, it should be greater than 1.3; (2) Breakend variants that cannot be assigned to one of the three classes were removed. (3) The SVs located in the unplaced contigs were excluded, and (4) all the SVs less than 50 bp and greater than 1 Mbp were removed.

### 2.6. Functional Annotation

The Ensembl Variant Effect Predictor (VEP) software [44] was implemented to annotate the gene content of SVs using the final VCF file post-filtration.

### 2.7. Gene Ontology (GO) Terms and Kyoto Encyclopedia of Genes and Genomes (KEGG) Analysis

The DAVID software was used to identify GO terms and KEGG pathways of the function candidate genes of SVs. In this study, we used the David 2021 version for GO and KEGG analysis (https://david.ncifcrf.gov/summary.jsp, accessed on 23 March 2022). The top 20 GO terms based on their *p*-values were visualized using the R package ggplot2 [36].

## 3. Results

### 3.1. Population Genetic Structure

**Figure 1:**
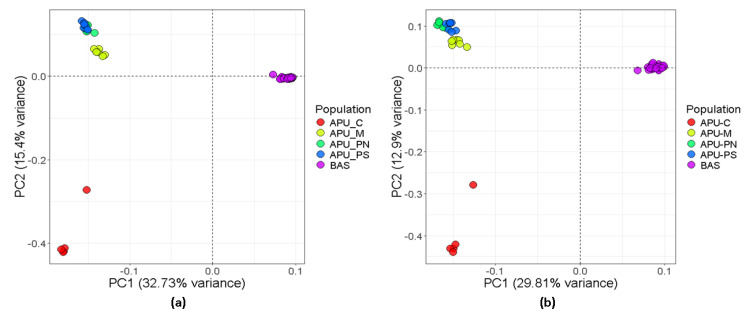
Principal component analysis (PCA) of the whole genome sequenced individuals. (**a**) PCA1 vs. PCA2 plot based on SNPs. (**b**) PCA1 vs. PCA2 plot based on structural variations. Apulian C (APU_C), Apulian M (APU_M), Apulian PN (APU_PN), Apulian PS (APU_PS), and Basilicata (BAS).

The PCA was conducted to assess the population’s genetic structure using SNP and structural variations (Figure 1). PCA1 revealed a clear separation of the BAS population from the APU_C, APU_M, APU_PN, and APU_PS populations. PCA2 showed that the APU_C population was distinct from the other Apulian groups, while the APU_M, APU_PN, and APU_PS populations were closely related, with overlapping between APU_PN and APU_PS. The PCA1 vs. PCA3 separated the APU_C, APU_M, APU_PN, and APU_PS populations (Supplementary File S2: Figure S1). These results demonstrate the high quality of the structural variation data.

**Figure 2:**
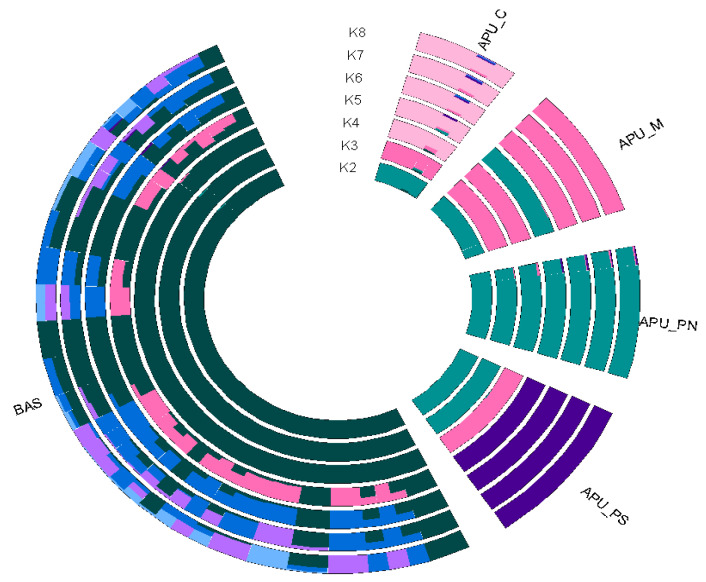
Admixture circle plot from K = 2 to 8 clusters for five Italian turkey populations. Apulian C (APU_C), Apulian M (APU_M), Apulian PN (APU_PN), Apulian PS (APU_PS), and Basilicata (BAS).

An admixture analysis with ancestral components (K) ranging from 2 to 10 was performed to further investigate population structure. The minimum cross-validation (CV) error identified K = 8 as the optimal number of ancestral populations (Supplementary File S2: Figure S2). The admixture analysis results were consistent with PCA findings (Figure 2). At K = 2, the BAS population was separated from the Apulian populations (APU_C, APU_M, APU_PN, APU_PS). At K = 3, the APU_C and APU_M populations were further separated from the APU_PN population. By K = 4, the APU_C population became distinct from the other Apulian groups, and the APU_PS population was separated from APU_PN. At K = 5, a substructure emerged within the BAS population, reflecting a more complex ancestral composition.

### 3.2. SV Discovery and Characterization

Whole genome sequences (WGSs) of 73 samples representing five turkey populations were analyzed to detect structural variants (SVs). The sequencing depth of these samples ranged from 6.01x to 15.13x, with an average depth of 11.44x (Supplementary File S1: Table S2). Across the five turkey populations, a total of 11,733 SVs were identified, comprising 6712 deletions, 2671 duplications, 1430 inversions, and 920 translocations. The summary statistics for the number and length of these SVs are provided in Table 1. Deletions were the most common type of SV identified. The highest number of deletions and duplications was observed in the 100–1000 bp length range, while most inversions were longer than 10,000 bp.

### 3.3. Distribution Across Chromosomes

**Figure 3:**
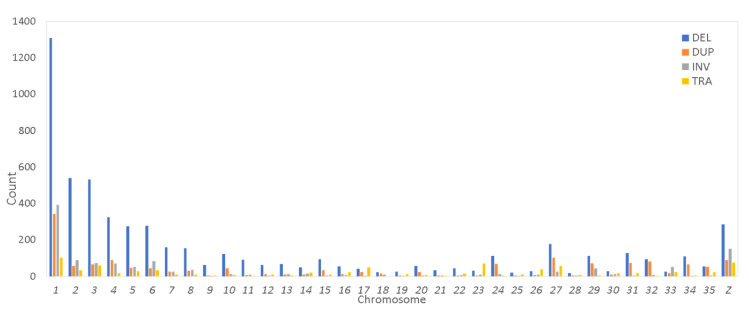
Distribution of discovered SVs per chromosome. Deletions (DELs), duplications (DUPs), inversions (INVs), and translocations (TRAs).

The distribution of SVs across the chromosomes was analyzed using whole genome sequence data. Most SVs were identified on the macro-chromosomes (Figure 3). Chromosome 1 exhibited the highest number of deletions, duplications, inversions, and translocations. Interestingly, the macro-chromosomes (with chromosome lengths > 50 Mb) had more inversions compared to duplications, except for chromosome 4. In contrast, the intermediate and micro-chromosomes generally displayed a higher number of duplications than inversions, with notable exceptions on chromosomes 8, 13, 14, 22, and 33.

**Figure 4:**
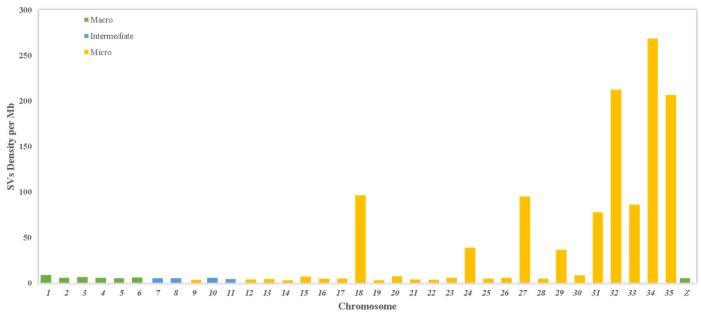
Chromosomal distribution of structure variations density on turkey genome. Chromosomes are split between macrochromosomes (>50 Mbp), intermediate chromosomes (>20 Mbp < 50 Mbp), and microchromosomes (<20 Mbp).

A comprehensive analysis of SV density and distribution across chromosomes was performed. The chromosomal distribution and density of all SVs are presented in Figure 4. The SV density in the micro-chromosomes was higher compared to the macro- and intermediate chromosomes in the turkey genome. Notably, micro-chromosomes 34, 32, and 35 exhibited the highest SV density within the turkey genome. Chromosome 18 also shows a high density of structural variants. This chromosome contains the MHC regions known to be enriched for structural variants.

### 3.4. Distribution of Structure Variations per Animal

The counts of each type of SV per animal are displayed in (Supplementary File S2: Figure S3) and detailed in (Supplementary File S1: Table S3). On average, the number of deletions per animal is 1452, with a range from 1299 to 1580. The mean number of duplications is 527, ranging from 468 to 557, while the average number of inversions is 95, with a range of from 79 to 110 (Supplementary File S1: Table S4).

### 3.5. Allele Frequency

**Figure 5:**
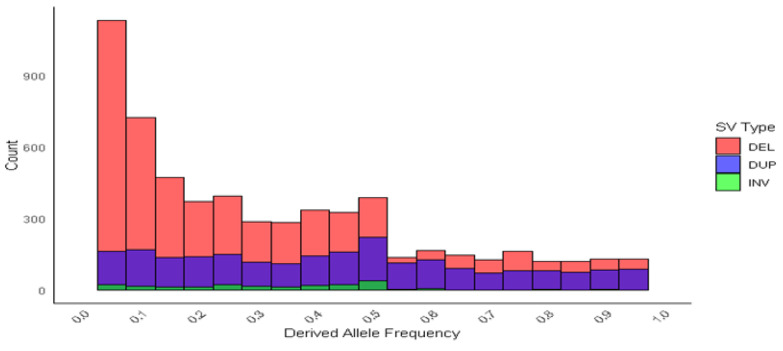
Derived allele frequency of structure variations. Deletions (DELs), duplications (DUPs), and inversions (INVs).

The deletions and duplications exhibited similar allele frequency spectra, both containing many rare variants. Notably, most of the inversions were rare, with only a small subset reaching an allele frequency of approximately 0.50 (Figure 5).

### 3.6. Functional Annotation

**Figure 6:**
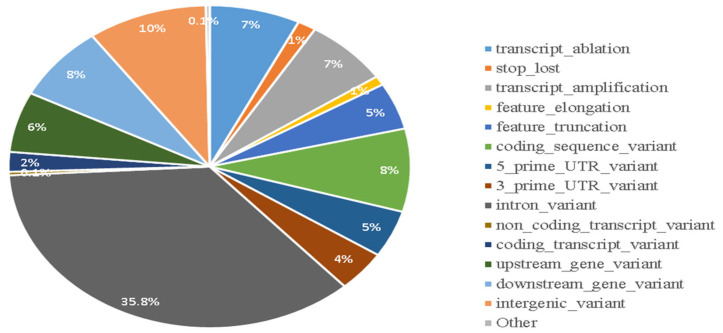
Distribution of structure variations situated in different genomic regions.

After applying post-filtration criteria, a total of 6918 SVs were reminded, comprising 5320 deletions, 1418 duplications, and 180 inversions (Supplementary File S1: Table S5). These SVs overlapped with 3387 genes and 7962 transcripts (Table S6). The VEP (Variant Effect Predictor) analysis predicted various consequences of these SVs: 35.8% were associated with intron variants, 9.6% with intergenic variants, 7.5% with downstream gene variants, and 7.3% with transcript ablations (Figure 6). Additionally, 8.3% of the SVs were found to be coding sequence variants in the turkey genome.

### 3.7. Gene Ontology and Kyoto Encyclopedia of Genes and Genomes

**Figure 7:**
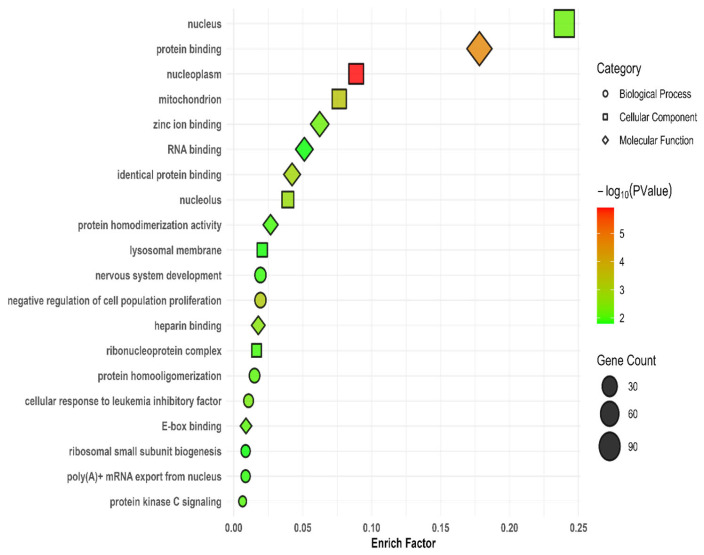
The gene ontology (GO) enrichment analysis of candidate genes. The top 20 enriched GO terms, ranked by *p*-value, were plotted. Biological processes, molecular functions, and cellular components are visualized by different shapes.

The 766 genes detected from the SVs annotation were used for functional analysis through GO and KEGG enrichment pathways. The GO enrichment analysis revealed 42 significant (*p* < 0.05) functional pathways (Supplementary File S2: Table S7). The top 20 GO terms, based on their *p*-values, are illustrated in Figure 7. Among these, the most significant terms are nucleoplasm (GO:0005654), protein binding (GO:0005515), mitochondrion (GO:0005739), negative regulation of cell population proliferation (GO:0008285), identical protein binding (GO:0042802), nucleolus (GO:0005730), and heparin binding (GO:0008201). KEGG enrichment analysis identified two pathways, one of which is significant: the calcium signaling pathway (mgp04020) (Supplementary File S2: Table S7).

## 4. Discussion

To further investigate the characteristics of structural variants (SVs) across five turkey populations, we conducted population genetic structure analyses. Notably, the BAS population was distinct from the Apulian populations, as shown in PCA1 vs. PCA2, while PCA1 vs. PCA3 separated the Apulian populations from each other in both SVs and SNPs. Together, our PCA results demonstrated that SV genotypes produced a population structure and ancestral components similar to those derived from SNP genotypes. The findings revealed that the genetic background of the BAS population differs significantly from that of the APU turkey populations. The close genetic relationship observed among APU populations may be linked to their geographic origins in southern Italy. These results were further supported by the ADMIXTURE analysis, which highlighted distinct background compositions for the APU and BAS populations.

Genome structural variations constitute a significant portion of genomic diversity and play critical roles in phenotypic variation and biological functions [21]. Unlike single nucleotide variations, which are relatively well studied, SVs remain largely underexplored due to limitations in detection methods [26,44]. In this study, we present the first comprehensive whole-genome SV landscape in Italian turkeys, which may be associated with traits such as growth and reproduction, providing valuable insights for future research. We detected 11,733 SVs across the turkey populations based on sequencing data. Among these, deletions accounted for the largest proportion of SVs (57.20%), followed by duplications (22.76%), inversions (12.18%), and translocations (7.84%). The prevalence of deletions aligns with previous studies, suggesting that non-allelic homologous recombination (NAHR) events may generate more deletions than duplications. Additionally, there may be a biological or algorithmic bias favoring the detection of deletions [45]. For example, deletions were more frequent than duplications and inversions in the bovine genome [26]. Zhang et al. [21] found 49,501 SVs in chickens, including 23,817 deletions, 3292 duplications, 20,847 insertions, 407 inversions, and 1138 translocations using PacBio data. Our SV length distribution analysis (Table 1) showed that most deletions (48.22%) and duplications (38.48%) ranged between 100 and 1000 bp, while most inversions (69.30%) exceeded 10,000 bp. All translocations measured between 1 and 50 bp. Consistent with other studies, SVs were not uniformly distributed across the genome [46]. For example, Upadhyay et al. [26] found that the average deletion length (~2.4 Kb) was significantly smaller than duplications (~29.9 Kb) and inversions (~46.4 Kb). Previous studies using next-generation sequencing technology to detect SVs in Xiang pigs [47], chickens [48], and cattle [15,49] identified only a few SVs larger than 10 Kb.

The influence of structural variants (SVs) on the genome and their distribution patterns across chromosomes can provide important insights into genetic diversity. In this study, the number of SVs per chromosome ranged from 1308 to 18. Macro-chromosomes exhibited the highest number of SVs, with deletions accounting for the majority, while duplications, inversions, and translocations made up a smaller proportion (Figure 3). Interestingly, micro-chromosomes displayed a higher density of SVs compared to macro-chromosomes, where structural variation was notably lower. In cattle, Boussaha et al. [25] found that Chr12 contained the highest percentage of SVs (approximately 7% of the total), followed by ChrX (5.5%) and Chr23 (5%). No clear relationship between SV types and chromosomal distributions was observed. For the macro-chromosomes (from Chr1 to Chr5), the proportion of total SVs was 21.7%, 16.2%, 9.8%, 7.6%, and 4.3%, respectively, in chickens [21]. Zhang et al. [21] also noted that SVs were more densely distributed on micro-chromosomes than on macro-chromosomes, with one SV every 2.94 kb on chromosome 30 and every 2.06 kb on chromosome 32. Similarly, Qiao et al. [24] observed that the highest number of SVs and their distribution in the sheep genome occurred on chromosomes 1, 2, and 3.

The range of SVs per animal was from 1887 to 2201, showing relatively little variation among individuals. In comparison, a study by Upadhyay et al. [26] identified a broader range of SVs per individual in cattle, from 2502 to 7164, highlighting greater differences across the cattle genome.

The allele frequency of deletions and duplications is skewed towards lower frequencies, with the majority falling between 0.0 and 0.5. This distribution is similar to findings in cattle, where Lee et al. [15] observed that deletions and duplications exhibited comparable allele frequency spectra, with many rare variants present. In particular, most duplications were uncommon, with only a few reaching an allele frequency of approximately 0.25. Similarly, [29] reported that, in pigs, the proportion of rare SVs (allele count, AC = 1 to 10; allele frequency, AF < 0.01) was higher than that of singleton SVs (AC = 1). Most SVs were classified as common (AC > 10, AF > 0.01), suggesting a lower level of diversity in these pig populations. This lower diversity in common SVs may contribute to the smaller percentages of singleton and rare SVs observed in these animals.

In this study, the most prevalent consequences for SVs in this study were intron variants (9994) coding sequence variants (2323), and transcript ablations (2033). In the cattle genome, 361 SVs caused ablation and 482 caused amplification, affecting entire transcript features [26]. Additionally, 790 SVs were found in the coding regions of the cattle genome. In total, 3078 SVs (2.4%) overlapped with 3080 (15.9%) protein-coding genes, according to Yang et al. [29]. This is much lower than the number of SVs found in noncoding regions (8174 SVs, 6.3%), intergenic regions (62,871 SVs, 48.2%), and introns (57,179 SVs, 43.8%, affecting 3307 or 17.1% of genes). Only 749 SVs (0.6%) were located within exons, mostly in the 3′ regulatory regions (3′-UTR) of 595 genes (0.5%), suggesting that SVs could potentially alter gene function by disrupting these regions.

Gene Ontology (GO) enrichment analysis showed that some SVs related to genes in the turkey populations were enriched in pathways for biological processes, molecular function, and cellular components. These pathways included nucleoplasm, protein binding, mitochondrion, negative regulation of cell population proliferation, and identical protein binding, while KEGG pathway enrichment revealed significant association with the calcium signaling pathway. Similarly, Qiao et al. [24] reported that specific SV-related genes were enriched in plasma membrane adhesion molecules and the calcium-signaling pathway of the GO and KEGG pathways, respectively, in Southdown sheep.

## 5. Conclusions

This study offers a deeper understanding of structural variations in turkeys and presents an SV catalog comprising 11,733 SVs identified through whole-genome sequencing of turkeys. The identified structural variations in turkeys may impact the function of associated genes, potentially influencing key production traits such as nucleoplasm, protein binding, mitochondrion, negative regulation of cell population proliferation, identical protein binding, and calcium signaling pathway. Therefore, our genome-wide SV catalog for turkeys provides valuable insights into the possible genomic basis of economically important traits in turkeys.

## Figures and Tables

**Table 1:** The length distribution of structural variations by type.

Length/Type	Deletions	Duplications	Inversions
0–50 bp	274	0	13
50–100 bp	1062	0	11
100–1000 bp	3237	1028	142
1000–10,000 bp	1581	984	273
>10,000 bp	558	659	991
Total	6712	2671	1430

## Data Availability

Whole genome data are available at https://www.ncbi.nlm.nih.gov/sra/PRJNA1197539, (accessed on 12 December 2024).

## Supplementary Materials

The following supporting information can be downloaded at https://www.mdpi.com/article/10.3390/ani15030339/s1: Figure S1: Principal component analysis (PCA) of the whole genome sequenced individuals. (a) PCA1 vs. PCA3 plot based on SNPs. (b) PCA1 vs. PCA3 plot based on structural variations. Apulian C (APU_C), Apulian M (APU_M), Apulian PN (APU_PN), Apulian PS (APU_PS), and Basilicata (BAS). Figure S2: Cross validation error. Figure S3: Distribution of structure variations per animal, (A) Deletions, (B) Duplications and (C) Inversions; Table S1: Detailed sample information on sequenced birds; Table S2: NGS data reads mapping; Table S3: Basic statistics of the different types of SVs identified in this study; Table S4: Summary statistics for SVs; Table S5: Post filtration structure variations detected in this study, in VCF format.; Table S6: Functional annotation of post-filtered SVs in this study; Table S7: GO terms and KEGG pathways of all SVs.
