# Supplementary material for: Genome Wide Identification of Structure Variations in Five Italian Turkey Populations
Source: Animals (Basel). 2025 Jan 24;15(3):339. doi: 10.3390/ani15030339 (PMC11816156; doi:10.3390/ani15030339)
Supplement: Supplementary file 1 [file animals-15-00339-s001.zip › Supplementary File S2_Animals-R1-final.pdf]

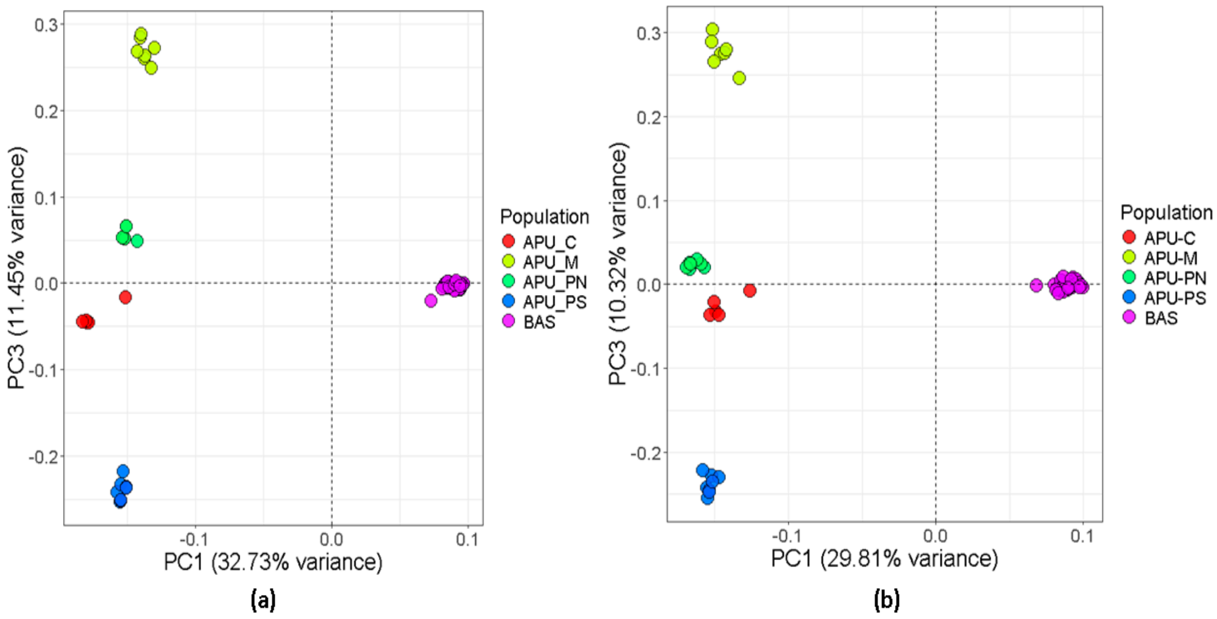

Figure S1. Principal component analysis (PCA) of the whole genome sequenced individuals. (a) PCA1 vs PCA3 plot based on SNPs. (b) PCA1 vs PCA3 plot based on structural variations. Apulian C (APU_C), Apulian M (APU_M), Apulian PN (APU_PN), Apulian PS (APU_PS), and Basilicata (BAS).

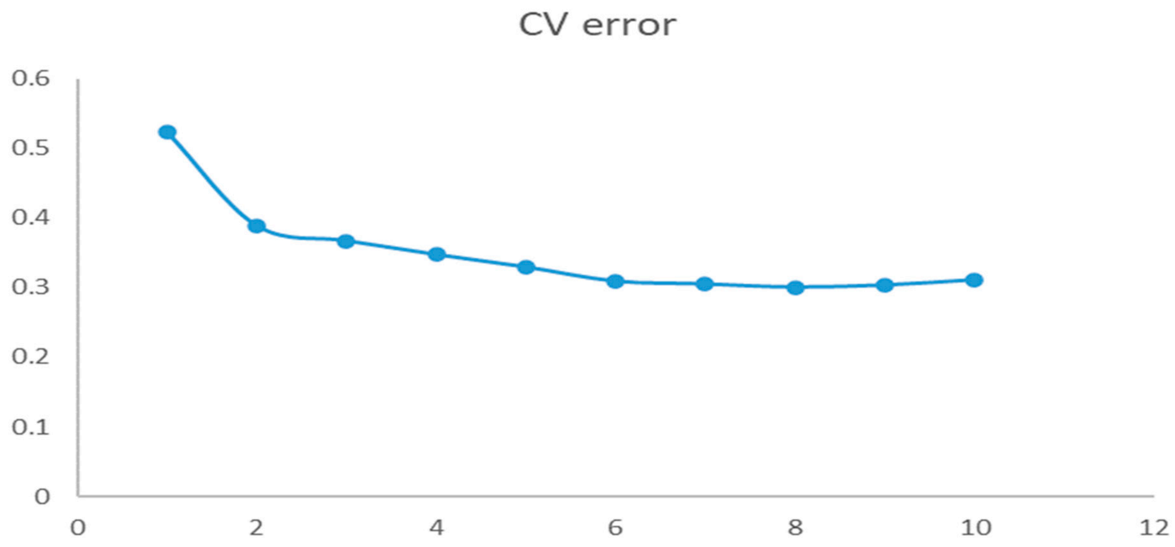

Figure S2. Cross validation error

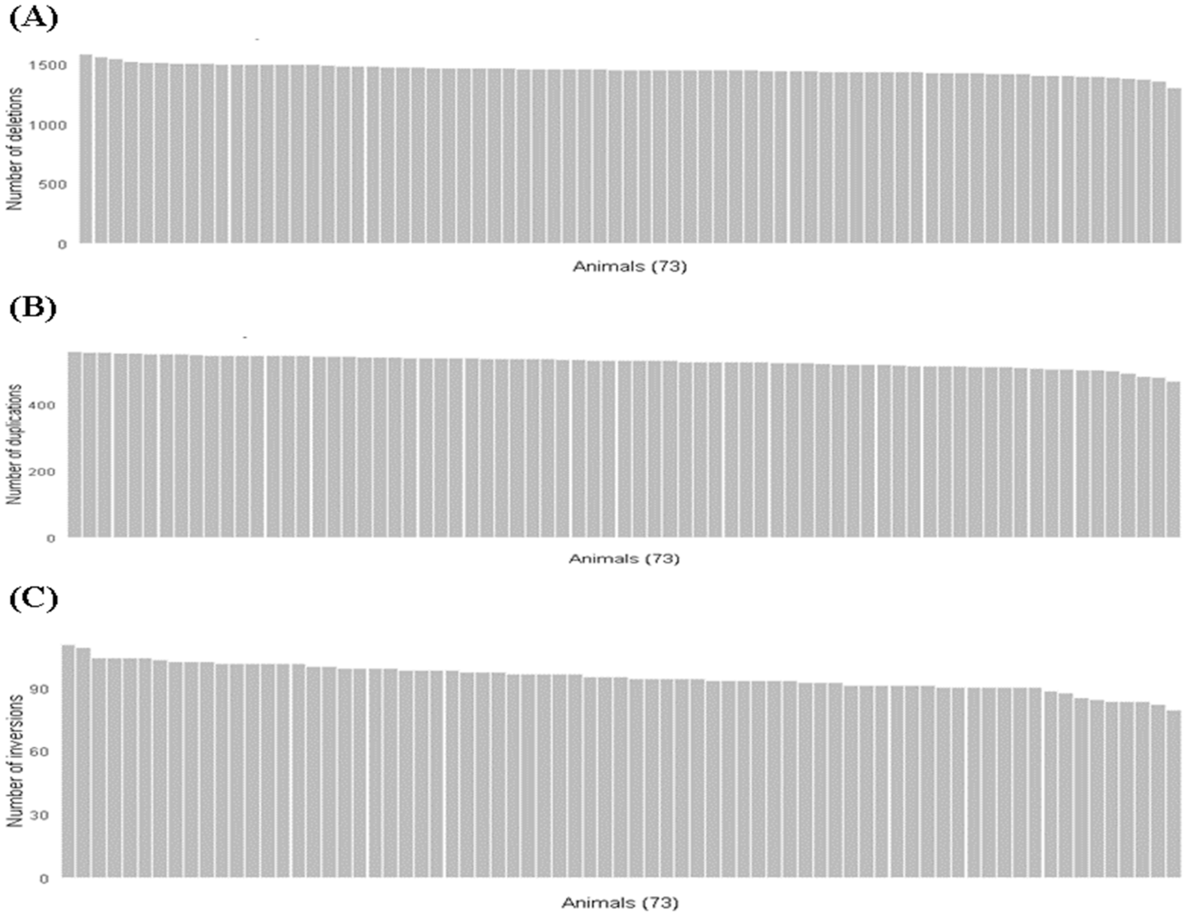

Figure S3. Distribution of structure variations per animal, (A) Deletions, (B) Duplications and (C) Inversions.
